# Acute Right Lower Abdomen in a Patient with a History of Gastrointestinal Stromal Tumor

**DOI:** 10.1155/2019/6091515

**Published:** 2019-02-21

**Authors:** Dabanjan Bandyopadhyay, Hugo J. R. Bonatti

**Affiliations:** ^1^University of Maryland Community Medical Group, Surgical Care, 500 Cadmus Lane, Easton, MD 21601, USA; ^2^Osteopathic University of Pikeville, KY, USA; ^3^Meritus Surgical Specialists, Hagerstown, MD, USA

## Abstract

**Background:**

Gastrointestinal stromal tumor (GIST) is an uncommon tumor of the GI tract usually seen in elderly patients, often difficult to diagnose because of the unspecific symptoms such as abdominal pain and fullness. Recurrent GIST may have an even more obscure clinical presentation.

**Case Report:**

A 44-year-old female with a history of surgically treated GIST presented to the emergency room complaining of acute onset sharp RLQ pain, nausea, and vomiting. Clinically, she had RLQ tenderness with rebound, her WBC was elevated, and CT scan showed signs of appendicitis and also soft tissue masses suspicious for malignancy. After contemplating treatment options including antibiotics and further workup, it was recommended to proceed with surgery. Laparoscopy showed a thickened appendix with nodular infiltration and multiple mass-like lesions in the RLQ not amendable to minimal invasive resection. From a lower midline incision, an open appendectomy and excision of multiple masses in the terminal ileum and in the abdominal wall were done. Narrow-based Meckel's diverticulum with multiple nodular lesions was also removed. Pathology identified appendicitis and serosal involvement of GIST in all specimens staining positive for CD68, CD117, and vimentin. The patient was started on imatinib and remained recurrence-free after 6 months.

**Conclusions:**

This case illustrates a rare presentation of acute symptomatic recurrent metastatic GIST. Our patient was unusually young, and GIST recurrence presented with acute RLQ pain suggestive for acute appendicitis and also involved Meckel's diverticulum. Surgical debulking followed by imatinib seems to be a reasonable approach in such cases.

## 1. Introduction

Gastrointestinal stromal tumor (GIST) comprises less than 1% of all GI tumors [1]. Histologically, they look similar to a smooth muscle cell tumor; however, they originate from the interstitial cells of Cajal (ICC). Approximately, 60% of GISTs originate from the stomach with the small bowel being the second most common site affected. Primary appendiceal GIST is extremely rare [2, 3]. The CD117 gene is positive in the majority of GISTs. This marker is used as a key indicator in diagnosis, and this is also the target of the tyrosinase kinase inhibitor (TKI) imatinib, which is used for systemic treatment in the neoadjuvant, adjuvant, and metastatic setting. Second-line treatment with newer TKIs, such as sunitinib and regorafenib, is available for imatinib-resistant GISTs, and new molecular-targeted therapies are on the horizon [4, 5]. GIST is usually seen in elderly patients who frequently present with nonspecific symptoms such as abdominal pain and abdominal fullness. Increasingly, GIST is an incidental finding on CT scan done for other indications. Treatment of the tumor is based on staging according to size and the mitotic index [6]. Surgical resection remains the first step in isolated lesions, with TKIs being powerful agents to control growth of the tumor [4, 6, 7]. GIST usually spreads intraperitoneally and to the liver, while lymph node involvement is rare.

Acute appendicitis may be treated with antibiotics alone; however, most surgeons still consider laparoscopic appendectomy the treatment of choice for most individuals [8]. CT scan is highly accurate in diagnosing appendicitis, and also, other pathologies in the case of RLQ pain may be detected. Primary, recurrent, and metastatic GIST may involve RLQ structures including the appendix and Meckel's diverticulum. If such a diverticulum is found incidentally during any abdominal surgery, it should be removed in order to prevent subsequent complications. Primary GIST in Meckel's diverticulum has been reported [9, 10], however not so in a recurrent GIST.

We herein report the rare case of recurrent metastatic GIST to both the appendix and Meckel's diverticulum in a patient presenting with RLQ pain and appendicitis on CT scan.

## 2. Case Report

**Figure 1:**
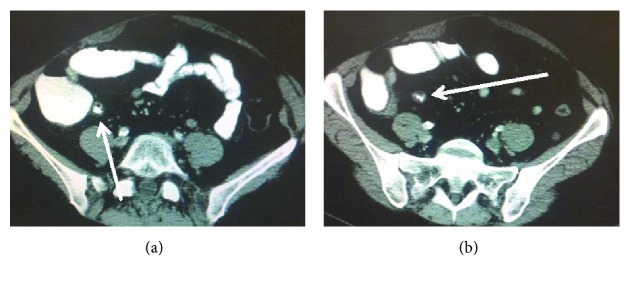
(a, b) CT scan: signs of appendicitis (stranding, diameter 9 mm), suspicious soft tissue masses.

A 44-year-old female presented to the emergency room with sudden onset sharp right lower quadrant pain, nausea, and vomiting. On physical exam, she was positive for McBurney's, Rovsing's, psoas, and obturator signs. Her white blood cell count was elevated at 16.5. CT scan showed signs of appendicitis as well as suspicious intraabdominal soft tissue masses (Figure 1). Five years earlier, she presented to a different hospital with diffuse abdominal pain, and CT scan identified a small bowel lesion; endoscopic biopsy showed GIST. She underwent SB resection without complications. No pathology was available, and according to the patient, no adjuvant chemotherapy was given; on her one-year follow-up CT scan, no evidence for tumor recurrence was found with no additional oncologic follow-up. She remained symptom-free for the next several years, until this episode.

**Figure 2:**
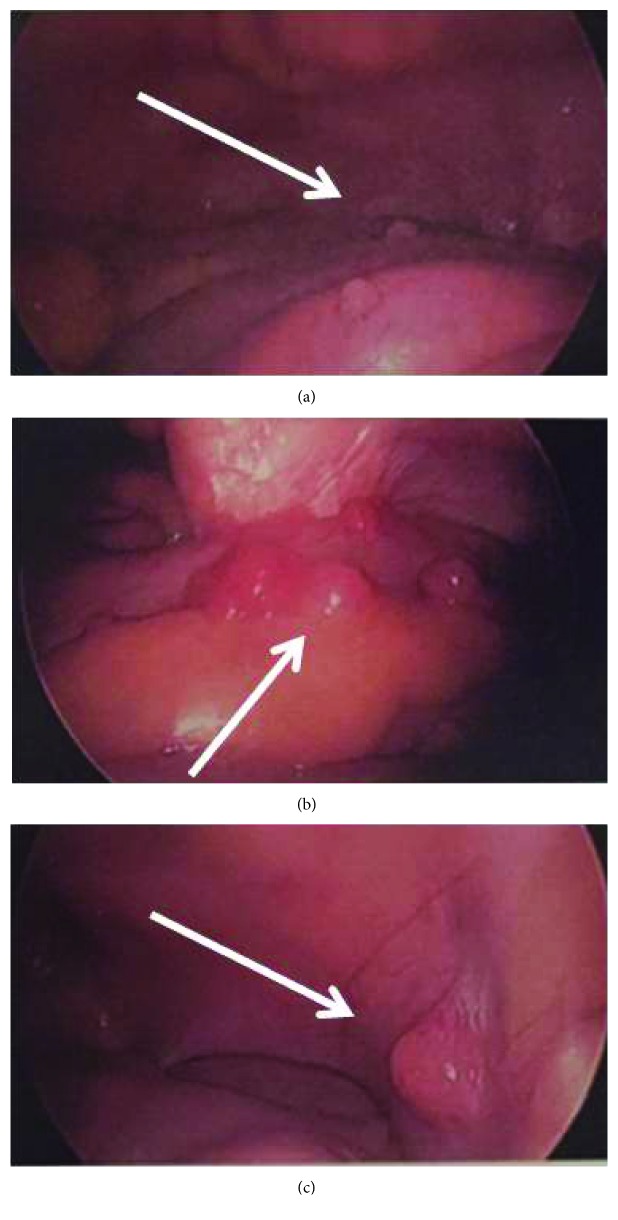
(a–c) Intraoperative findings: nodules on the appendix, terminal ileum, and visceral and parietal peritoneums.

Secondary to the CT findings suggestive for appendicitis, nonoperative management and further workup were contemplated. The RLQ masses were most concerning for recurrent GIST, and after discussion with the patient, indication for surgery was made. On diagnostic laparoscopy, the appendix wall was found thickened and acutely inflamed with nodular lesions. In addition, multiple up to 2 cm in diameter nodules on the terminal ileum and the parietal peritoneum of the anterior and lateral abdominal walls were visualized (Figure 2). One larger nodule in the right pelvis could not be mobilized. The liver was found to be free of any lesions. Due to the volume of the separate masses and inability to mobilize the large right pelvic nodule out of the pelvis, the case was converted to laparotomy. A lower midline incision was made. The appendix was mobilized, the mesoappendix was secured using a stapler, and the appendix was resected at the base and handed off for pathology. All visible nodules were then removed from the peritoneum, and a partial resection of the terminal ileum was performed. When the small bowel was run from the terminal ileum to the jejunum, at 100 cm from the TI, narrow-based Meckel's diverticulum with multiple nodular lesions was found. The diverticulum was resected at the base using a stapler. On pathology, acute appendicitis was seen; however, also serosal involvement of metastatic GIST was noted in all specimens including the appendix and the Meckel diverticulum. The tumor stained positive for CD68, CD117, and vimentin.

The patient had an uneventful recovery and was discharged from the hospital on postoperative day five. The patient was started on imatinib, and at the 6-month interval, she was doing well without any complaints; CT scan showed no evidence for recurrent GIST. She was then lost to follow-up when she moved.

## 3. Discussion

This is a rare presentation of GIST, in an unusually young patient with simultaneous GIST lesions to the appendix and a Meckel diverticulum amongst other intraabdominal sites. The patient presented with RLQ pain, elevated WBC, and CT scan findings suggesting acute appendicitis. At time of presentation to our ER, the patient was not aware of possible recurrent GIST; however, such pathology was immediately considered.

Operative management remains the primary treatment modality for low-grade GIST tumors. For high-risk tumors, the recurrence rate after surgical resection alone remains high [11] with recurrent GIST having the same risk profile as metastatic tumor, for which the first line of treatment is imatinib [11]. Surgical options for recurrent GIST are limited, but tumor debulking has remained a good option. Imatinib alone in this setting provides a low complete response rate but is associated with a good disease control rate [11]. The current recommendation for initial treatment of recurrent GIST in a stable patient is imatinib. In symptomatic patients such as ours, surgery should be considered to reduce the tumor burden and to treat and/or prevent surgical complications such as obstruction, perforation, and hemorrhage [12]. Nonoperative management and further workup were contemplated; however, we opted for laparoscopy given the patient acute clinical symptoms and imaging and laboratory findings. Even in the setting of recurrent metastatic GIST, acute appendicitis may still develop independently although this is probably extremely rare. The appendix showed signs of acute inflammation on laparoscopy, and pathology confirmed the presence of appendicitis in addition to GIST involvement. It is impossible to determine if this was an independent finding or if this was triggered by spread of the GIST to the appendix. Once it was determined that the procedure could not safely be finished in a minimally invasive fashion, a laparotomy was made with extensive tumor debulking and resection of incidentally found Meckel's diverticulum which was also involved in the metastatic disease. Pathology confirmed GIST in all specimens; the tumor stained positive for CD117, and imatinib was started. GIST tumors are often fragile, and care should be taken in resection—as tumor rupture or bleeding leads to an increased risk of recurrence [11]. Reduction of the tumor burden followed by administration of a TKI has remained the accepted strategy for metastatic GIST necessitating surgical intervention [5, 7]. Prognosis is acceptable with many patients surviving for more than 5 years [6].

In patients with a history of GIST who present with abdominal symptoms, recurrent disease should be considered. Metastatic and primary GISTs may present similar to acute appendicitis [2, 3]; even perforation of GIST within Meckel's diverticulum has been described [10]. Nevertheless, spread of GIST both to the appendix and Meckel's diverticulum is a very rare condition with as yet undefined guidelines for surgical intervention.
